# Simulation of 316L Stainless Steel Produced the Laser Powder Bed Fusion Process

**DOI:** 10.3390/ma16247653

**Published:** 2023-12-15

**Authors:** Ľuboš Kaščák, Ján Varga, Jana Bidulská, Róbert Bidulský

**Affiliations:** 1 Department of Technology, Materials and Computer-Aided Production, Faculty of Mechanical Engineering, Technical University of Košice, Letná 9, 04002 Košice, Slovakia; jan.varga@tuke.sk; 2 Department of Plastic Deformation and Simulation Processes, Institute of Materials and Quality Engineering, Faculty of Materials, Metallurgy and Recycling, Technical University of Košice, Vysokoškolská 4, 04200 Košice, Slovakia; 3 Bodva Industry and Innovation Cluster, Budulov 174, 04501 Moldava and Bodvou, Slovakia; director@biic.sk; 4 Advanced Research and Innovation Hub, Budulov 174, 04501 Moldava and Bodvou, Slovakia

**Keywords:** metal additive manufacturing, Simufact Additive, mechanical properties, cryogenic conditions

## Abstract

Additive manufacturing is increasingly being used in the production of parts of simple as well as complex shapes designed for various areas of industry. Prevention of errors in the production process is currently enabled using simulation tools that have the function of predicting possible errors and, at the same time, providing a set of information about the behaviour of the material in the metal additive manufacturing process. This paper discusses the simulation processes of 316L stainless steel produced using the laser powder bed fusion (L-PBF) process. Simulation of the printing process in the Simufact Additive simulation program made it possible to predict possible deformations and errors that could occur in the process of producing test samples. After analysing the final distortion already with compensation, the simulation values of maximum deviation −0.01 mm and minimum −0.13 mm were achieved.

## 1. Introduction

In order to reduce the weight of parts, additive manufacturing technology is increasingly used in a wide range of industries [1,2,3,4,5,6,7,8,9,10,11,12]. The most commonly used technology is laser powder bed fusion technology (LPBF). Due to the complexity of the process, LPBF technology involves more than 100 processing parameters, the essential ones being scanning speed, laser power, coating thickness, distance between subsequent laser transitions, and scanning strategy [2]. Parts produced using this technology are accompanied by distortion as well as residual stress, which, as a result, has a negative impact on the required dimensional and geometric accuracy of the parts [3]. Due to the thermal processes that take place in the metal printing process (heating and cooling cycles), large thermal gradients occur, as a result of which high thermal stresses are created [4,5].

It is also necessary to expose the manufactured parts after the LPBF process to additional operations in the form of heat treatment or surface treatment. This is because the essence of the LPBF process is also nonequilibrium thermal conditions, as well as extremely fast cooling rates and a sharp thermal gradient. As a result, there are high thermal residual stresses in the part, which must be eliminated or minimised [13,14].

The roughness parameters obtained using LPBF technology reach high values of Ra to an extent of 6–9 μm, which can be observed on vertical surfaces. One way to reduce these values is to use surface treatment, sandblasting, or polishing [15,16]. Another option to avoid high roughness is the application of machining technology in the form of a finishing operation. Due to the complexity of LPBF parts, 5-axis machining is a suitable way to achieve it. Therefore, it is necessary to consider the basic data that play an essential role in the process of metal additive manufacturing as early as in the process of designing the shape of a part. In addition to basic parameters such as laser power, layer thickness, and scanning speed, it is also necessary to consider information for building the model, such as fill pattern, density, part orientation, number of layers, etc. [17]. When questioning the functional aspect of manufactured parts with this technology, it is necessary to know the characteristics of the manufactured part, for which several tests can be used. Some of the most widely used tests used to evaluate material properties are mechanical tests. Several researchers evaluated the impact of process parameters on the mechanical properties of parts, using mainly tensile testing for evaluation purposes [18,19,20].

Dababneh and Taheri dealt with the impact of an interruption of the printing process on the mechanical properties of parts made using metal additive technology [21]. The intermittent process resulted in negative material and mechanical properties in the examined part. In their research, Debroy et al. [22] described the problems encountered in the process of manufacturing LPBF technology, focusing on the residual stresses that occurred in the manufacturing process, porosity, and evaporation of elements. They also expanded their research to include findings describing the texture of grains obtained when using this method of production. Pasternak et al. [23] investigated the individual stages of the shear mechanism in the powder sintering process, focusing on changing the structure of the surface layer of inhomogeneous materials.

The analysis of the influence of the filling percentage used in the production of samples on mechanical properties was dealt with by Hsueh et al. [18]. Johnson, by performing tensile tests, demonstrated the direct influence of the density of the part filling during production on yield strength and the Young’s modulus of elasticity [24]. Also, the influence of the orientation of the construction of the part on the bending properties was investigated by Suwanpreecha and Manonukul [25], and another interesting research was conducted by Caminero et al. [26], who evaluated the influence of the nozzle diameter on the geometric accuracy in printing metal parts from 316L stainless steel.

In their research, some authors dealt with the application of the lattice structure in the production process and the evaluation of the products’ mechanical properties. Gu et al. [27] evaluated the isotropic elastic properties of lattice structures based on various experimental studies. Limpitipanich et al. [28] and Fongsamootr et al. [29] investigated 3D lattice structures and their behaviour in bending tests. Bjørheim and Lopez [30], on the other hand, carried out a mechanical tensile test on produced samples made of 17-4PH materials; however, these were processed using the BMD method.

Paraschiv et al. [31] investigated the tensile strength of additively manufactured undersized samples made of IN 625 material and compared them with standard samples. They used round sample shapes produced using the LPBF method as samples, while for the final form they used non-standard-shaped samples due to material consumption and production time. This resulted in lower tensile strength and yield strength compared to the values obtained for standard samples. The tensile properties of austenitic stainless-steel material, specifically 21-6-9, were investigated by Neikter et al. [32], who dealt with the optimisation of LPBF process parameters with respect to grain size, density, and hardness. In their experiment, they dealt with the influence of room temperature and a temperature of 1023 K, as well as the influence of the orientation of the structure during construction.

Process simulation can be useful in predicting the output of a given orientation and support strategy. However, it should be noted that specialised process simulation programs are still relatively rare and are often developed due to the recent introduction of laser powder bed fusion as a leading manufacturing technology.

The task of optimisation is to find parameters for the production process that allow the production of parts with minimised errors, the necessary density, and reduced surface roughness while achieving the desired properties of the part, taking into consideration the increasing production capacity. Parameter optimisation is necessary due to constant changes; even when the same type of material is used, manufacturing technology might have changed. In the past, a large part of research and analysis dealing with the influence of input parameters on the additive manufacturing process was carried out through various studies and numerical models. They included approaches based on the principle of FEM, which included not only a thermomechanical model but also the method of inherent deformation [33,34,35]. These numerical models are used in simulation software to analyse the manufacturing process (injection moulding, forming, joining) and thus prevent errors in the manufacturing process [36].

Kaščák et al. [37] introduced the Simufact Additive program, in which they predicted the behaviour of the material in metal additive manufacturing. They focused on the input parameters of the process and the generation of the support structure in defining the critical surface angle, which was set at 45°. In the simulation mode, the volume fraction was compared, as were the deviations obtained from the standard method of generating support material and using the support optimisation function. By using the support material optimisation function, a different distribution of support was achieved compared to the conventional method, obtaining the following values:

− generation of support without optimisation Σ volume 71,875.6 mm^3^
− generation of support with optimisation of Σ volume 42,693.7 mm^3^

The purpose of applying these simulation tools to the metal additive manufacturing process is to understand the process itself, as it differs from classical 3D printing, where the manufacturing process consists of adding molten plastic layer by layer [38,39]. It is also necessary to consider the size of the elements, which is one of the decisive factors in the process of metal printing, when using a simulation tool designed to predict the behaviour of the material in the process and the deviation [40].

Despite the larger number of simulation tools designed for metal additive manufacturing, the Simufact Additive program was used for the purpose of the experiment, which, with its performance and scalable software solution, is suitable for simulating additive manufacturing processes based on metals. The great advantage of these simulation tools used for the additive manufacturing process is not only the wide possibilities of entering input parameters but also the ability to control stress states as well as the geometry of the part. Last but not least, microstructure control is made possible on the basis of the volume of the part using process maps [41]. The importance of data processing and data exchange with the support of processing of the most widely used formats in the field of CAD systems and various simulation programs was dealt with by Kurylo et al. [42], who emphasized the importance of the data for several production technologies.

The possibilities of current simulation software make it a useful tool in additive manufacturing. However, due to the complexity of the metal additive manufacturing process, this area is still not fully explored. The behaviour of individual layers in the construction process with respect to different process input parameters was investigated by Majeed et al. [43].

The rate of cooling of individual layers, morphology, and melt dimensions was analysed in their research by Hu et al. [44], applying the finite element method (FEM).

Zhou et al. [45] created an FE model that examined the temperature profile during the LPBF process and residual stress fields.

Very interesting was the research conducted by Pitassi et al. [46], who created the FE model based on the simulation of the thermal behaviour of a melting pool formed during the process. Another model characterised by a lattice was analysed by Kovaleva et al. [47], where the result was the use of contact surfaces between individual particles, making it possible to analyse thermal conductivity. Dai et al. [48], on the other hand, explored how the multilayer aspect of additive manufacturing affects molten pool dynamics, cooling rates, crystal sizes, microstructure morphology, microhardness, and residual stress types. The accumulated residual thermal stress arises from repeated thermal cycles in distinct solidified layers. Typically, tensile and compressive stresses are comprehensively present in the ultimately solidified layers, exerting a significant influence on microhardness.

Products made using metal printing technology are intended for various uses, depending on the mechanical properties obtained. The properties of products obtained using LPBF metal printing technology, such as mechanical properties, material properties, quality of powders, or material structure, are currently well known, which is reflected in several publications. However, these were mostly observed in normal operating conditions [49,50]. The missing link is information or knowledge of the behaviour of materials in environments with cryogenic temperatures, as well as the influence of cryogenic temperatures on the mechanical properties of the material [51].

Therefore, the novelty of this paper is to provide more information not only about the use of the LPBF process in the field of simulation but also with regard to the tensile properties of 316L stainless steel at cryogenic temperatures.

According to Villa and Somers [52], research into the influence of cryogenic temperatures on material microstructure and tensile properties is insufficient. In order to find out if a material is suitable for use at cryogenic temperatures, it is necessary to conduct research into its properties in such conditions.

Further research on the influence of heat treatment on materials has been carried out in this area. In their research, Stornelli et al. [53] analysed the effect of heat treatment on the hardness and microstructure of maraging steel class 300 using the L-PBF process. The result of the measurement was a hardness value of 583 HV obtained from ageing at 490 °C for 6 h. In their further research, di Schino and Stornelli [54] evaluated the conditions for achieving the best properties of magnetising alloys, focusing their research on the microstructure and texture of 6.5% FeSi steels.

This research aimed to predict errors that could occur in the production process using the simulation program Simufact Additive, whose functions provide a wide range of possibilities in the field of process analysis of metal additive manufacturing technology. The parameters obtained from the simulation were also applied to the real production process.

## 2. Materials and Methods

For the purpose of the experiment, a material in the form of powder was chosen, namely 316L stainless steel powder (manufacturer EOS, Krailling, Germany). Its primary alloying constituents after iron are chromium (16–18%), nickel (10–12%), and molybdenum (2–3%), with small (<1%) amounts of silicon, phosphorus, and sulphur. The individual samples intended for the tensile test were produced using LPBF technology on the EOSINT M270 Dual Mode machine (manufacturer EOS, Krailling, Germany). The input parameters of the production process were those listed in Table 1, which also served as input data for the simulation process in the Simufact Additive 2022 program.

A static tensile test was conducted on samples under three different temperature conditions: 298 K, 77 K, and 4.2 K. The MTS100 Landmark device (MTS, Eden Prairie, Minnesota, MN, USA), outfitted with a cryostat and extensometer, was employed for the tests. A minimum of three specimens for each condition underwent testing in accordance with the ASTM E8M standard. The cooling process to liquid nitrogen and helium temperatures was facilitated using a cryostat with a vacuum shield.

For the purpose of the experiment, the samples intended for production were divided into two groups:

− The first group of samples, designated as “as-built”;
− A second group of samples, designated as “stress-relieved”, which was subjected to an additional operation after the manufacturing process to reduce the stress generated in the part during the metal printing process. For the purpose of this heat treatment, a temperature of 673 K was applied for 1 h, and stress mitigation was realised in a vacuum furnace type TAV MiniJet HP 235 (TAV, Caravaggio, Italy) at a vacuum of 9 × 10^−3^ mbar. Using a low temperature, it was not necessary to apply an Ar backfill due to the degassing of the elements. More information in detail is given in our previous papers related to mechanical properties and cryogenic phenomena [55,56,57,58].

## 3. Results

### 3.1. Evaluation of the Analysis of the Printing Process in the Simufact Additive 2022 Simulation Program

**Figure 1:**
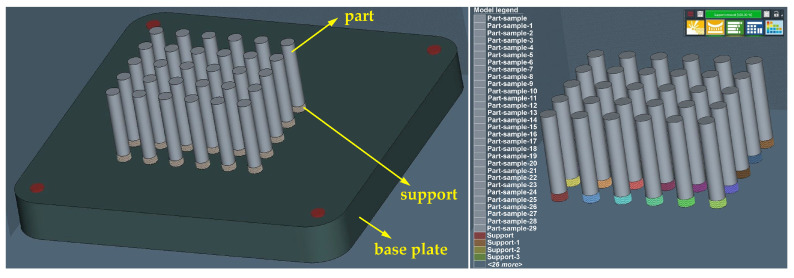
Display of test samples and supporting material in a simulation environment.

Since the production of samples for the tensile tests was planned to be 30 pcs, the same number was used for the simulation process. After defining the orientation of the construction of test samples and selecting the supporting material, we received an initial view of the environment as shown in Figure 1.

**Figure 2:**
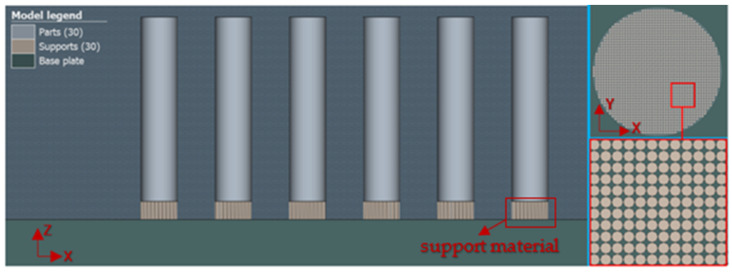
Display of support material geometry in the simulation area.

The choice of geometry for the construction of the support material was based on the simplicity of the shape of the part in the form of simple cylinders (see Figure 2). A closer view of the shape of the support material is given in the upper right corner of Figure 2.

**Figure 3:**
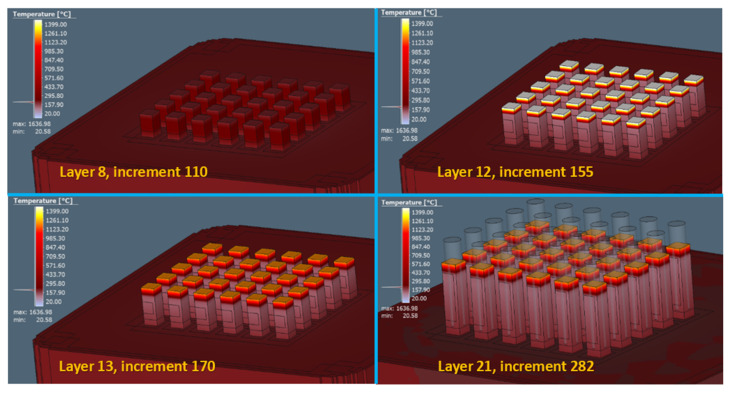
Display of gradual temperature transfer in the simulation process in a specific layer.

For simulation and verification of the functionality of the process of producing test samples using the LPBF method, input parameters according to Table 1 were implemented in the simulation area. Setting these parameters should allow us to predict possible errors that could occur in the production process. From the results of the volume fraction, 41,101 voxel elements, 54,749 nodal points, and a total of 28 layers for the construction process were obtained. A sample of the display of a gradual temperature transfer in a simulation process relative to a specific layer is shown in Figure 3.

The metal additive manufacturing process is closely related to a distortion factor that cannot be avoided in the manufacturing process. It is the result obtained by heating a smaller amount of material to the melting point on an otherwise cooler layer.

**Figure 4:**
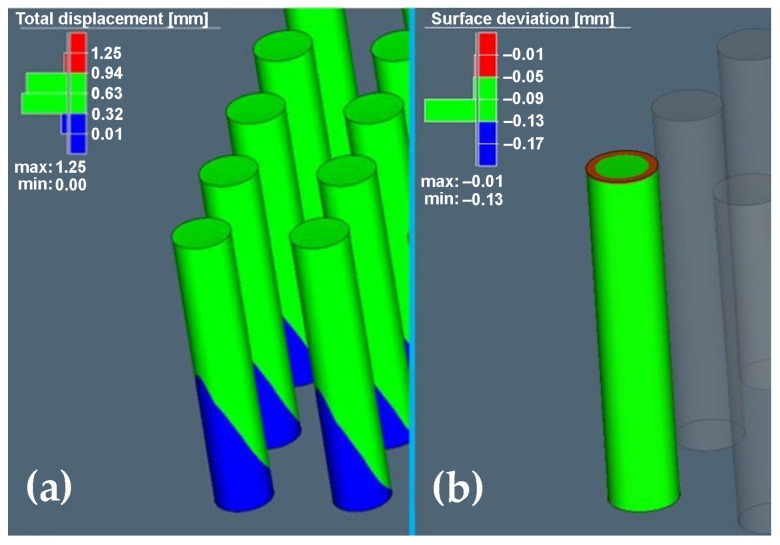
Display of final distortion: (**a**) without compensation; (**b**) with compensation.

The final distortion without compensation is shown in Figure 4a, where the maximum deviation shown in red reached 1.25 mm and the minimum deviation value without compensation was 0.01 mm. The areas rendered green ranged from 0.32 mm to 0.94 mm. The obtained results did not show any deviations on the samples corresponding to the red area, but despite the fact that it was a simple part, and in order to later produce samples with the required accuracy and also with regard to geometry, the calculation of compensation for the given geometry was carried out as shown in Figure 4b.

After analysing the final distortion with compensation, the simulation values of maximum deviation −0.01 mm and minimum −0.13 mm were achieved.

### 3.2. Evaluation of the Tensile Test

**Figure 5:**
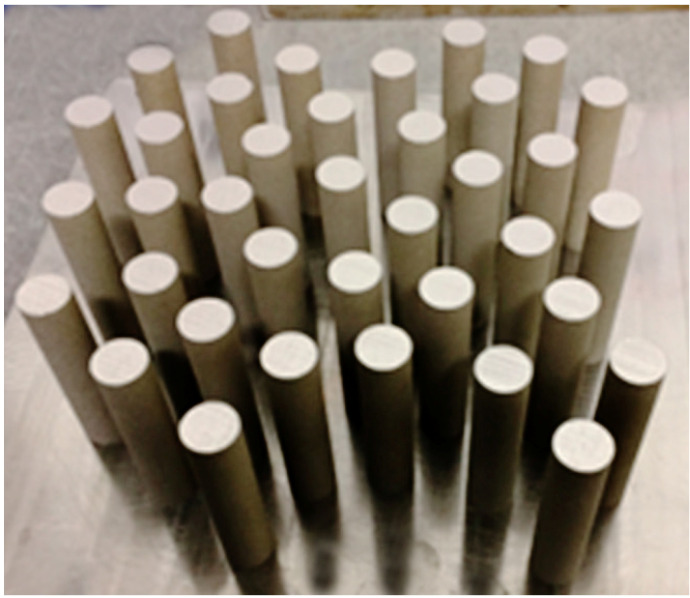
As-built samples just after the L-PBF printing process.

Based on the optimisation of the LPBF process by means of numerical simulation, samples were produced—see Figure 5.

Both the elongation values, represented by UE and TE, and the reduction in area (RA) serve as indicators of a material’s ductility. However, an analysis of the results in Table 2 reveals that they do not exhibit the same pattern as temperatures decrease. The RA decreases with declining temperatures, whereas both UE and TE exhibit a peak at 77 K. The elongation values in Table 2, measured with an extensometer and reproduced in samples under two conditions (as-built and stress-relieved), help mitigate the impact of potential errors. UE represents elongation before localised necking in the sample. After necking at cryogenic temperatures (77 K and 4.2 K), further plastic deformation is minimal, resulting in a negligible difference between TE and UE, below 1%.

Samples tested at room temperature experience lower YS, leading to necking after just 4% elongation. However, elongation continues until reaching a TE of 35% rupture, with a 31% difference between TE and UE. Deformation after necking primarily occurs in the neck area, explaining the highest RA measurement at 298 K. At cryogenic temperatures, the measured RA almost corresponds to that at the onset of necking. At 77 K, the sample exhibits UE higher than 50% along the entire gauge length, while at 4.2 K, such deformation ranges between 28% and 36%. The RA measurement is performed at the thinnest part of the gauge length. The substantial difference between UE and TE, 5% vs. 35%, respectively, indicates that after necking, the material continues to deform plastically before breaking. This observation is supported by the RA value of almost 50%. Samples tested at room temperature undergo progressive cross-sectional shrinkage, breaking after reaching a TE of approximately 35%. In contrast, samples tested at cryogenic temperatures experience a decrease in RA value that is not coupled with a decrease in elongation; instead, elongation significantly increases (56% at 77 K and 28% at 4.2 K).

From a general point of view, it is well established in the literature that austenitic steels, such as 316L, show transformation-induced plasticity characteristics (TRIP); such behaviour is supposed to be responsible for the increase in UTS observed with decreasing temperature (Table 2). In addition, a toughening effect is associated with twinning, while the extremely fine grain size provided by LPBF is another factor affecting both toughness and strength. The explanation for increased UTS at cryogenic temperatures has to be searched within the interaction of such factors, competing with the reduction in strength due to the development of pores and the presence of defects induced by the manufacturing technology itself. Consequently, deformation twinning can lead to significant ductility but does not result in a high hardening rate of AM 316L. Due to the still low number of studies on microstructures after deformation, it is still unclear why AM 316L has a good elongation, which is reported to be as good as that made using other processes according to Manfredi [59], despite some residual porosity and the high residual stresses in AM samples according to Calignano [60].

At the moment, the literature has only a few papers focused on the characterisation of the tensile properties of L-PBF 316L in cryogenic conditions [61,62,63]. Also, the mechanical properties concerning the fractographic evaluation were mentioned in detail in our previous papers [55,56,57]. Our research team deals with microstructure in detail in [58].

## 4. Discussion

The Simufact Additive simulation program could be used to simulate the additive metal manufacturing process. Simulating the printing process in Simufact Additive made it possible to predict possible deformations and errors that could occur in the process of producing test samples. Based on the results achieved by defining input parameters in the simulation process, no violations or errors were found that would be critical for the production process or weaken the geometry of the part. Therefore, the input parameters applied in the simulation were also used for the actual production of test samples.

As Gao et al. pointed out in their analysis [64], wastage of building and supporting material is frequent in the AM process due to the trials and errors and multiple repetitions that can occur during the manufacturing process. Hence, for the analysis and prediction of errors in the real production process, the production of test samples was carried out with the support of a simulation tool, which is highly beneficial for both basic and applied research.

**Figure 6:**
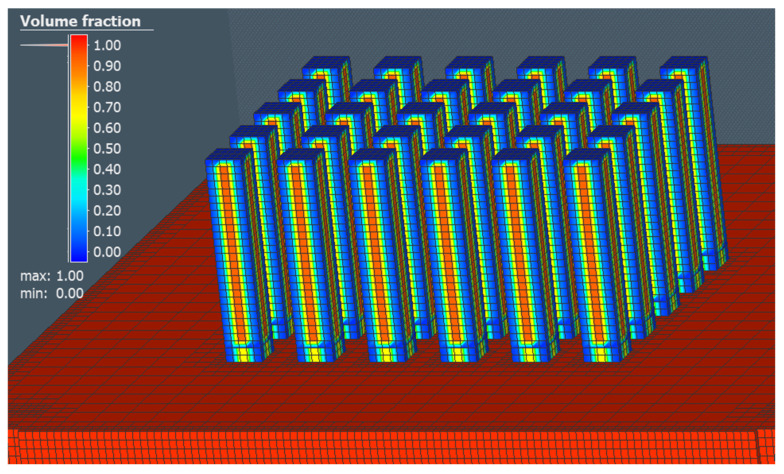
Display of the volume fraction of a part in the production of 30 pcs of test samples.

To discuss the volume fraction of a part, based on our results, it was possible to confirm the ease of creating a volume representation of the model based on voxel elements, as Tiede et al. carried out in their analysis [65]. In our case, the defined size of the element with a value of 2 mm for all directions X, Y, and Z allowed us to further analyse the temperature transfer in a particular layer as well as the distortion of parts. The value of 2 mm was chosen in relation to the simple geometry of the part as well as the speed of the calculation time. The display of the volume fraction in the production of 30 pcs of test samples in the simulation area is shown in Figure 6.

**Figure 7:**
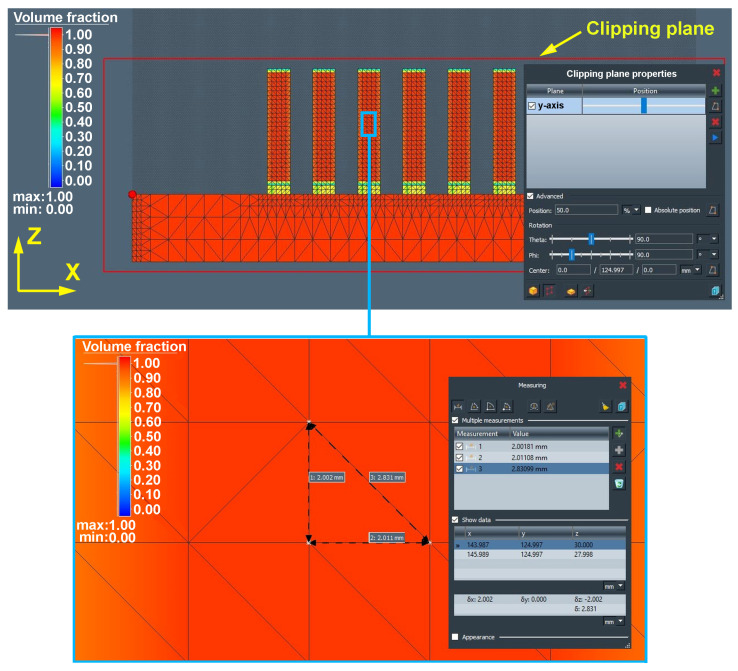
Display of the volume fraction of a part in a section.

In contrast to the results obtained in the research conducted by Cho et al. [66], where the volume fraction of a part was achieved only on the basis of local calculation, i.e., calculation in a specific plane, the simulation tool Simufact Additive enabled us to obtain a volume representation of the model in all three planes, thus allowing us to verify the quality of the network (Figure 7).

Since the input 3D models for additive manufacturing are represented by polygonal networks and, according to Ganesan and Fadel [67], should be multifaceted and waterproof, it is possible to confirm the suitability of using Simufact Additive simulation software for additive simulation purposes since the network of triangles showed no errors, intrinsic intersections, overlaps, gaps, or cracks that could not only disrupt the simulation calculation but also cause process instability and failure in the production process phase.

The display of the volume fraction in a section rendered sufficient network quality in the form of a colour gamut (red) at value 1, which showed no need to adjust the size of an element.

## 5. Conclusions

This research advocates for a comprehensive approach to designing components intended for production through LPBF. This involves integrating functional and structural requirements with the demands of additive manufacturing and the final finishing stage. To optimise the LPBF process, numerical simulation means were used, which predicted the behaviour of 316L stainless steel in the additive manufacturing process.

Based on the results of the numerical simulation using Simufact Additive, real samples were produced, eliminating the need to examine large numbers of such samples.

Further research should be supplemented with an assessment of deviations in shape and dimensional accuracy by the method of digitisation of DIC.

## Figures and Tables

**Table 1:** Process parameters for sample production.

Power [W]	Scanning Speed [mm/s]	Layer Thickness [µm]	Hatching Distance [mm]	Building Platform Temperature [K]
195	800	20	0.09	354

**Table 2:** Mechanical properties of 316L after tensile test at different temperatures.

Samples	TT (K)	YS [MPa]	UTS [MPa]	UE [%]	TE [%]	RA [%]
As-built	298	499 ± 12.1	564 ± 15.4	4 ± 2.7	35 ± 3.4	49 ± 2.1
	77	726 ± 17.7	1083 ± 36.2	53 ± 7.4	53 ± 7.9	23 ± 3.4
	4.2	802 ± 23.5	1246 ± 42.6	35 ± 3.2	36 ± 4.1	16 ± 1.1
Stress-relieved	298	500 ± 31.2	565 ± 19.4	5 ± 3.1	18 ± 6.2	48 ± 4.5
	77	730 ± 17.6	1080 ± 29.3	55 ± 6.4	56 ± 8.5	24 ± 3.1
	4.2	805 ± 32.4	1200 ± 34.2	27 ± 4.7	28 ± 3.2	15 ± 2.3

(YS—yield strength; UTS—ultimate tensile strength; UE—uniform elongation; TE—total elongation; RA—reduction in area).

## Data Availability

The research data can be obtained from the authors.
